# Neurotrophic Factors NGF, GDNF and NTN Selectively Modulate HSV1 and HSV2 Lytic Infection and Reactivation in Primary Adult Sensory and Autonomic Neurons

**DOI:** 10.3390/pathogens6010005

**Published:** 2017-02-07

**Authors:** Andy A. Yanez, Telvin Harrell, Heather J. Sriranganathan, Angela M. Ives, Andrea S. Bertke

**Affiliations:** 1 Department of Biology, Virginia Tech, Blacksburg, VA 24061, USA; andy2195@vt.edu; 2 Multicultural Academic Opportunities Program, Virginia Tech, Blacksburg, VA 24061, USA; tharr014@odu.edu; 3 Department of Population Health Sciences, Virginia-Maryland College of Veterinary Medicine, Virginia Tech, Blacksburg, VA 24061, USA; hjscrugg@vt.edu; 4 Department of Biomedical Sciences and Pathobiology, Virginia-Maryland College of Veterinary Medicine, Virginia Tech, Blacksburg, VA 24061, USA; amives@vt.edu

**Keywords:** herpes simplex virus, HSV1, HSV2, primary neurons, neurotrophic factors, latency, reactivation

## Abstract

Herpes simplex viruses (HSV1 and HSV2) establish latency in peripheral ganglia after ocular or genital infection, and can reactivate to produce different patterns and frequencies of recurrent disease. Previous studies showed that nerve growth factor (NGF) maintains HSV1 latency in embryonic sympathetic and sensory neurons. However, adult sensory neurons are no longer dependent on NGF for survival, some populations cease expression of NGF receptors postnatally, and the viruses preferentially establish latency in different populations of sensory neurons responsive to other neurotrophic factors (NTFs). Thus, NGF may not maintain latency in adult sensory neurons. To identify NTFs important for maintaining HSV1 and HSV2 latency in adult neurons, we investigated acute and latently-infected primary adult sensory trigeminal (TG) and sympathetic superior cervical ganglia (SCG) after NTF removal. NGF and glial cell line-derived neurotrophic factor (GDNF) deprivation induced HSV1 reactivation in adult sympathetic neurons. In adult sensory neurons, however, neurturin (NTN) and GDNF deprivation induced HSV1 and HSV2 reactivation, respectively, while NGF deprivation had no effects. Furthermore, HSV1 and HSV2 preferentially reactivated from neurons expressing GFRα2 and GFRα1, the high affinity receptors for NTN and GDNF, respectively. Thus, NTN and GDNF play a critical role in selective maintenance of HSV1 and HSV2 latency in primary adult sensory neurons.

## 1. Introduction

Following infection and replication in the epithelium, herpes simplex viruses 1 and 2 (HSV1 and HSV2) gain entry into sensory and autonomic neurons innervating the site of infection [1,2,3,4]. In some neuronal populations, the viruses replicate and kill the neurons. In other types of neurons, the viruses do not replicate and instead establish latency, from which they can periodically reactivate to cause recurrent disease. Each virus has shown a preference for latent infection in specific populations of neurons, which differ between HSV1 and HSV2 [5,6,7]. Approximately half of HSV1 latent sites are detected in a population of sensory neurons identified as Fe-A5+ neurons, but few latent sites are detected in neurons recognized by the monoclonal antibody KH10 (KH10+) or isolectin GS-IB4 (IB4+), due to selective HSV1 replication in KH10/IB4+ neurons, which kills them [5,7]. In contrast, HSV2 preferentially establishes latency in KH10/IB4+ neurons and selectively replicates in Fe-A5+ neurons, resulting in Fe-A5+ neuronal cell death and minimal latent sites detectable in Fe-A5+ neurons [6,8]. Thus, HSV1 and HSV2 preferentially replicate and establish latency in different populations of sensory neurons (Table 1) [6,7,9,10]. However, these preferred sites of latency only account for about half of the latent sites and it is not clear whether the viruses reactivate from these populations, raising the question as to which types of neurons support both latency and reactivation. Numerous studies have shown that both viral and neuronal factors play a role in regulating lytic vs. latent infection. How the mechanisms differ for HSV1 and HSV2, promoting either lytic infection or establishment of latency in different populations of neurons, remain unclear.

Sensory trigeminal (TG) and dorsal root ganglia (DRG) are comprised of a heterogeneous population of neurons. Each subpopulation is characterized by a distinct pattern of peripheral and central projections, as well as distinct neurotrophin requirements during development [11]. While sympathetic ganglia consist of a more homogeneous population of neurons with respect to neurotrophin requirements, receptor diversity is apparent and axonal projections differ substantially [12,13,14]. Approximately 80% of sensory neurons and all sympathetic neurons require nerve growth factor (NGF) for survival, differentiation and axonal targeting during embryonic development [15]. NGF continues to regulate sympathetic neuron morphology and survival into adulthood [16]. NGF is also required for sensitivity to sensory stimuli and neuron-specific gene expression in certain mature sensory neuronal populations [17]. However, about half of the small sensory neurons in the TG and DRG downregulate NGF receptors (TrkA) during the first three weeks after birth and are no longer regulated by NGF in maturity. These neurons, most of which are labeled by the lectin IB4, switch their dependence to glial cell line-derived neurotrophic factor (GDNF) postnatally [15]. The GDNF family ligands, including GDNF and neurturin (NTN), signal though a multicomponent receptor complex consisting of the ligand-binding glycosylphosphatidylinositol (GPI)-anchored coreceptors and the signaling component receptor tyrosine kinase RET [18]. GDNF and NTN preferentially bind and signal through GFR alpha 1 and GFR alpha 2 (GFRα1 and GFRα2), respectively, but can also bind the other receptor with lower affinity [18]. Ligand binding results in activation of multiple signaling pathways, including extracellular signal-related kinase/cAMP response element binding protein (ERK/CREB), phosphatidylinositol 3-kinase (PI3K)/AKT, p38 mitogen activated protein kinase (MAPK), and c-Jun N-terminal kinase (JNK), promoting cell survival, differentiation, and neuronal regulation [18]. Fe-A5+ neurons, in which HSV1 preferentially establishes latency, are a subset of TrkA+ NGF-responsive neurons [19]. In contrast, IB4+ neurons express RET in maturity, suggesting that the neurons in which HSV2 preferentially establishes latency are responsive to GDNF family neurotrophic factors, rather than NGF [15].

Viral reactivation occurs when the relationship changes between the host neuron and the latent HSV episome in response to a variety of environmental triggers, such as exposure to UV light, fever, stress, and nerve trauma [20,21]. Previous studies with HSV1 in embryonic and neonatal sympathetic and sensory neurons showed that withdrawal of NGF was sufficient to cause reactivation of the virus, leading to the conclusion that NGF was important for maintaining HSV1 in a latent state, and furthermore, that the strength and duration of signaling through the TrkA receptor tyrosine kinase (RTK)/Akt pathway controls HSV1 latency in cultured SCG neurons [22,23,24]. However, adult sensory neurons are no longer dependent on NGF for survival, although this neurotrophin continues to support the function of some sensory neurons in adulthood [25]. Additionally, HSV1 and HSV2 preferentially establish latency in different populations of neurons, suggesting that different factors may maintain latency in different types of neurons [6]. Furthermore, HSV2 preferentially establishes latency in IB4+ neurons, which switch their dependence from NGF to GDNF during postnatal development [15,26], suggesting that NGF may not be responsible for maintaining HSV2 latency. Finally, establishment of HSV1 and HSV2 latency in Fe-A5+ and KH10/IB4+ neurons only accounts for about half of the latent sites, and it is not clear whether the viruses reactivate from these neurons, suggesting the existence of a third population of sensory neurons that support reactivation from latency.

Using adult primary neuronal cultures from sensory and sympathetic murine ganglia, we investigated HSV1 and HSV2 latency dependence on neurotrophic factor signaling in mature, differentiated neurons. We determined that different neurotrophic factors have different impacts on HSV1 and HSV2 replication in adult sensory neurons. NGF contributes to the maintenance of HSV1 latency in primary adult sympathetic neurons, correlating with previous studies in embryonic sympathetic neurons [22,27], but not in primary adult sensory neurons. HSV1 and HSV2 are maintained in a latent state in their respective neuronal populations by different neurotrophic factors.

## 2. Results

### 2.1. NGF and GDNF Deprivation Induces Reactivation in Sympathetic Neurons

**Figure 1:**
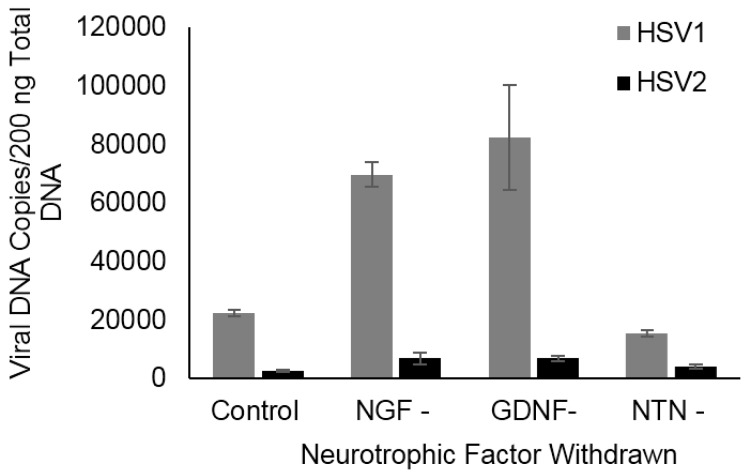
Effect of neurotrophic factor (NTF) deprivation on HSV reactivation in adult SCG neurons. Nerve growth factor (NGF) and glial cell line-derived neurotrophic factor (GDNF) deprivation induced reactivation of HSV1, but not HSV2, shown by significantly increased viral DNA copies 24 h after removal of individual NTFs and addition of their respective neutralizing antibodies after 7 days of acyclovir (ACV)-induced latency, compared to control neurons maintained in all three neurotrophic factors (*p* = 0.005 and 0.01).

Previous studies showed that NGF deprivation efficiently induced HSV1 reactivation from cultured sympathetic neurons derived from embryonic mouse superior cervical ganglia (SCG) [22,24]. Although these neurons were dissected from embryos, neurons continue to mature in vitro and were of postnatal age when infected and when reactivation was triggered. Although sympathetic neurons become much less dependent on NGF for survival as they mature in culture or in vivo, they remain dependent on NGF for normal neuronal morphology and metabolic function [28]. Therefore, we sought to determine if NGF deprivation induced reactivation of HSV1 and HSV2 in cultured mouse sympathetic neurons derived from adult mice. After establishment of experimental latency with acyclovir (ACV) for seven days, withdrawal of NGF and addition of anti-NGF antibodies to fully deplete NGF in the media induced HSV1 reactivation in the cultured adult sympathetic neurons, compared to cultures maintained with NGF, GDNF, and NTN, as shown by a significant increase in viral DNA (Figure 1, *p* = 0.005). Since sympathetic neurons can also be maintained with other neurotrophic factors, we also deprived latently infected adult sympathetic neurons of either GDNF or NTN. While GDNF withdrawal induced reactivation of HSV1 comparably to NGF deprivation (*p* = 0.01), NTN deprivation had no effect on HSV1 (Figure 1). In contrast, none of the neurotrophic factors efficiently induced reactivation of HSV2 (Figure 1). There were no significant differences between control neurons maintained in all three NTFs, with and without ACV removal.

### 2.2. NTN Deprivation Enhances HSV1 and HSV2 Acute Viral DNA Replication in Adult Sensory Neurons

**Figure 2:**
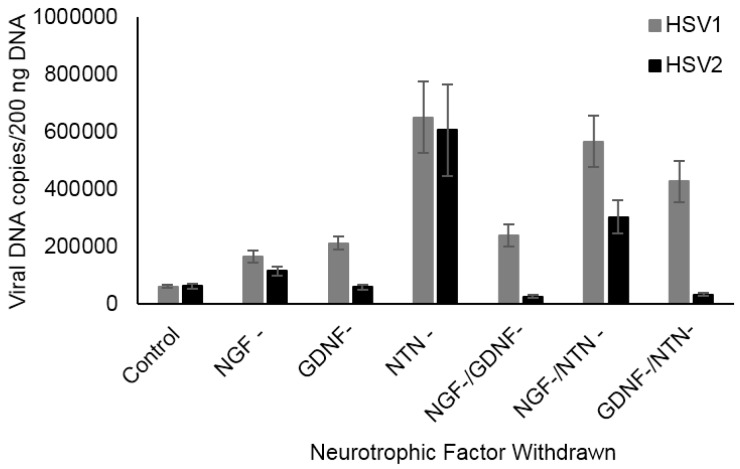
Effect of NTF deprivation on acute viral replication in adult murine sensory neurons. Deprivation of NGF, GDNF, or NTN, either individually or in combinations, significantly increased HSV1 replication in adult sensory trigeminal (TG) neuronal cultures (*p* = 0.031, 0.007, 0.039, 0.003, 0.004, <0.001), compared to control neurons maintained in NGF, GDNF, and NTN, shown by increased viral DNA loads quantified by qPCR. In contrast, only NTN deprivation and NGF/NTN deprivation significantly enhanced HSV2 replication in adult sensory TG neuronal cultures (*p* = 0.034, 0.009).

HSV1 and HSV2 preferentially establish latent infection in different types of sensory neurons, as a result of their inability to lytically infect those specific types of neurons (which typically results in cellular death) [5,9]. Therefore, neurotrophic factors may modulate productive infection in specific types of sensory neurons, leading to preferential establishment of latent infection from which the viruses can later reactivate. To assess neurotrophic factor (NTF) effects on acute replication of HSV1 and HSV2 in adult sensory neurons, we deprived cultured TG neurons of either NGF, GDNF, or NTN during acute infection for 24 h. Loss of NGF or GDNF signaling increased the detected number of HSV1 viral genome copies 2 fold (*p* = 0.031) and 3.5 fold (*p* = 0.007), respectively, compared to infected control neurons maintained with all three neurotrophic factors (Figure 2), but had no effect on HSV2 replication. In contrast, NTN deprivation during acute infection increased both HSV1 and HSV2 replication 10-fold, compared to infected controls (Figure 2, *p* = 0.039 and 0.034, respectively). Although GDNF and NTN signal through their preferred GDNF family receptors alpha 1 and 2 (GFRα1 and GFRα2), respectively, they can also signal through the other receptor with lower affinity. In addition, NGF, GDNF, and NTN fulfill some complementary functions in adult sensory neurons. Therefore, we also deprived infected neurons of two neurotrophic factors simultaneously. Deprivation of both NGF and GDNF enhanced HSV1 replication comparable to deprivation of either NGF or GDNF alone, but had no effect on HSV2 (Figure 2, *p* = 0.003). Similarly, deprivation of both GDNF and NTN enhanced HSV1 replication, but not HSV2 (Figure 2, *p* < 0.001). In contrast, deprivation of both NGF and NTN enhanced both HSV1 and HSV2 replication (Figure 2, *p* = 0.004 and 0.009, respectively). Thus, disruption of any of the three neurotrophins during acute infection is able to enhance HSV1 replication, but the presence of NTN produces an inhibitory effect on HSV1 replication during acute infection. The presence of NTN also inhibits acute replication of HSV2, although simultaneous disruption of both NTN and GDNF appears to produce an interaction that negates this effect.

### 2.3. HSV1 and HSV2 Reactivation Is Induced in Adult Sensory Neurons by Deprivation of Different Neurotrophic Factors

**Figure 3:**
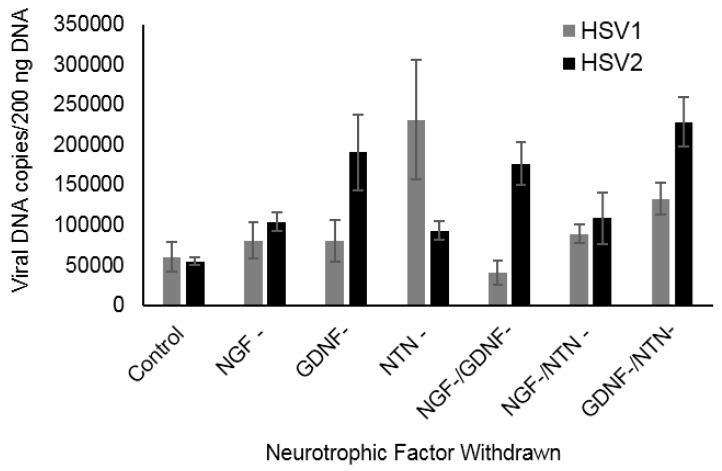
Effect of NTF deprivation on reactivation in adult sensory neurons. Deprivation of NTN induced reactivation of HSV1, either alone (*p* = 0.034) or in combination with GDNF deprivation (*p* = 0.012), compared to control neurons maintained in all three NTFs, shown by significantly increased HSV copy numbers 24 h after withdrawal of NTFs and addition of antibodies. There was no significant difference between NTN- and GDNF-/NTN- for HSV1. Deprivation of GDNF induced reactivation of HSV2, either alone (*p* = 0.042) or in combination with NGF (*p* = 0.040) or NTN (*p* = 0.015) deprivation, compared to controls.

To determine if deprivation of NGF, GDNF, or NTN induces reactivation of HSV1 and HSV2 in adult sensory neurons, we established experimental latency in the presence of ACV and all three neurotrophic factors for seven days. Removal of ACV alone from control neurons did not produce a significant increase in viral DNA compared to those with ACV remaining in the media, showing that the cultures were latently infected. After withdrawal of ACV and individual neurotrophic factors, NTN withdrawal induced HSV1 reactivation, shown by significantly increased viral DNA (Figure 3, *p* = 0.034). Furthermore, deprivation of NTN in combination with GDNF deprivation also induced HSV1 reactivation (*p* = 0.012), although other combinations did not reactivate HSV1. Deprivation of both NGF and GDNF had no effect, suggesting that the continued presence of NTN in the media suppressed HSV1 reactivation. When NTN and NGF were both withdrawn, the presence of GDNF in the media appeared to provide a suppressive effect on HSV1 reactivation, suggesting that GDNF may partially compensate for the loss of NTN. Thus, NTN maintains HSV1 latency, although GDNF can partially compensate for loss of NTN signaling. In contrast, GDNF deprivation induced HSV2 reactivation in primary adult sensory neurons, either alone or in combination with NGF or NTN deprivation (Figure 3, *p* = 0.042, 0.040, 0.015), showing that GDNF signaling is a requirement to maintain HSV2 latency in adult sensory neurons.

### 2.4. HSV1 and HSV2 Reactivate from Different Populations of Neurons

**Figure 4:**
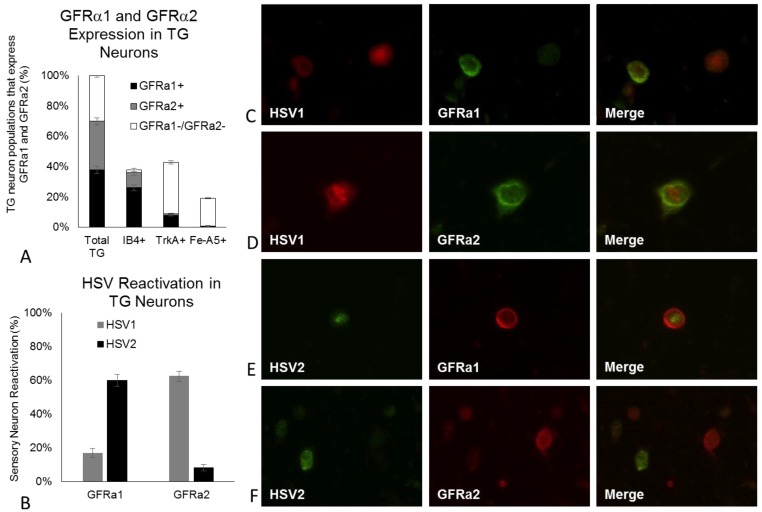
GFRα1 and GFRα2 receptor expression. (**A**) Approximately 1/3 of sensory TG neurons express GFRα1 (37.7%) and GFRα2 (32.1%), while the remaining neurons do not express either GFR (30.2%). 68.9% of IB4+ neurons express GFRα1 and 26.3% express GFRα2. 17.9% of TrkA+ neurons express GFRα1 but only 2.8% express GFRα2. GFRα1 was detected in 2.5% of Fe-A5+ neurons, which are a subset of TrkA+ neurons, but GFRα2 was detected on less than 1% of these neurons; (**B**) HSV1 and HSV2 reactivate from different subpopulations of neurons, with HSV1 reactivating preferentially from GFRα2+ neurons and HSV2 reactivating preferentially from GFRα1+ neurons, the high affinity receptors for NTN and GDNF, respectively; (**C**–**F**) Representative images of reactivating HSV1 and HSV2, observed as RFP and GFP expression in neuronal nuclei, co-localizing with GFRα2 and GFRα1 immunofluorescence, respectively.

Our results show that HSV1 reactivation can be induced by loss of NTN signaling, while HSV2 reactivation is induced by loss of GDNF signaling in adult mouse sensory neurons. Based on the combinatorial deprivations however, it is possible that interactions may be occurring downstream of receptor binding, if receptors are present on the same neurons, or that different neuronal populations may have variable levels of sensitivity to the NTFs. We found that approximately one third of adult sensory TG neurons express GFRα1, the high affinity receptor for GDNF (37.7%), or GFRα2, the high affinity receptor for NTN (32.1%) (Figure 4A). The remaining third does not express either GFR. IB4+ neurons represented 37.2% of the TG neurons and of these, 68.9% expressed GFRα1 and 26.3% expressed GFRα2. TrkA+ neurons represented 42.8% of the cultured neurons; GFRα1 was detected in 17.9% of TrkA+ neurons but only 2.8% expressed GFRα2 detectable by immunofluorescence. We identified 19.1% of the cultured TG neurons as Fe-A5+ neurons, which are a subset of TrkA+ neurons. However, few of these neurons expressed detectable GFRα1 (2.5%) or GFRα2 (0.6%) (Figure 4A). Since GDNF and NTN preferentially signal through GFRα1 and GFRα2, respectively, but can also cross over to signal through the other receptor with lower affinity, we sought to determine if HSV1 and HSV2 reactivate from the GFRα1 or GFRα2 positive sensory neurons. Following removal of ACV and all three NTFs from neurons latently infected with HSV1-VP26-RFP and HSV2-VP26-GFP, which express red fluorescent protein (RFP) and green fluorescent protein (GFP) during productive infection, HSV1 and HSV2 reactivated preferentially in different sensory neurons (Figure 4B). Although 16.9% of HSV1 reactivations were detected in GFRα1+ neurons, 62.4% occurred in GFRα2+ neurons, expressing the NTN high affinity receptor (Figure 4B). In contrast, 60.0% of HSV2 reactivations occurred in GFRα1+ neurons, expressing the GDNF high affinity receptor, while only 8.2% of HSV2 reactivations were detected in GFRα2+ neurons (Figure 4B). Nearly two-thirds of HSV1 reactivations, observed as RFP expression in neuronal nuclei, co-localized with GFRα2 immunofluorescence (Figure 4D) rather than with GFRα1 (Figure 4C), while the opposite was observed for HSV2, observed as a GFP expression in GFRα1+ neurons (Figure 4E) instead of GFRα2+ neurons (Figure 4F).

## 3. Discussion

Previous studies showed that HSV1 and HSV2 reactivated in response to NGF deprivation in embryonic cultured sympathetic and sensory neurons [22,23,24]. Our results support and extend the previous reports, showing that NGF deprivation, and also GDNF deprivation, induces HSV1 reactivation in adult sympathetic neurons. In addition, we show that neurotrophic factor deprivation does not reactivate HSV2 in adult sympathetic neurons, suggesting that alternative mechanisms may induce HSV2 reactivation in these neurons. However, in adult sensory neurons, NTN and GDNF, rather than NGF, are important in the maintenance of HSV1 and HSV2 latency.

During postnatal development, a portion of sensory neurons downregulates expression of the high-affinity NGF receptor TrkA and begins expressing GDNF family receptors (GFRs), effectively switching their dependence from NGF to glial family neurotrophic factors, including GDNF and NTN. Although 80% of embryonic neurons in rodent and human DRG express the NGF receptor TrkA, half of these neurons downregulate TrkA postnatally and by adulthood, only 40%–50% of adult rat and human DRG neurons express TrkA and remain responsive to NGF [15,29,30]. Those that downregulate TrkA can be labeled by the lectin IB4, begin postnatal expression of GFRs along with the associated signaling tyrosine kinase Ret, and lose their responsiveness to NGF. HSV1 preferentially establishes latency in Fe-A5+ neurons, which are a subset of TrkA+ sensory neurons responsive to NGF, while HSV2 preferentially establishes latency in KH10/IB4+ neurons, dependent on GDNF family neurotrophic factors [6,7]. However, these populations harbor only about half of the latent HSV1 and HSV2 reservoirs. The remaining half are distributed among several different populations of sensory neurons, including those that express GFRs and are responsive to GDNF and/or NTN. Because the IB4+ populations downregulate TrkA expression to undetectable levels and are no longer responsive to NGF [15,26,29], we reasoned that NGF was unlikely to be responsible for maintaining viral latency within these IB4+ adult neurons. By depriving our infected neuronal cultures of individual neurotrophic factors, we identified NTN-responsive neurons as an important population of neurons for HSV1 latency and reactivation, and further identified GDNF-responsive neurons as important for HSV2 latency and reactivation. Thus, adult sensory neurons responsive to NTN and GDNF are the clinically relevant sensory neuronal populations from which HSV1 and HSV2, respectively, reactivate to cause recurrent disease in response to loss of neurotrophic factor support. Previous studies have reported that sensory neurons express either GFRα1 (GDNF receptor) or GFRα2 (NTN receptor) in adult mouse TG and DRG, as well as adult human DRG [30,31,32,33,34], suggesting that HSV1 and HSV2 reactivate from different populations of sensory neurons. A previous study also reported the existence of a subgroup of adult mouse IB4+ neurons that co-express both GFRα1 and GFRα2, as well as a small population of neurons (3%) that co-express TrkA and GFRα2 [34], which we also found. Given the apparent interaction when we deprived our HSV1-infected neurons of combinations of NTFs, we cannot rule out the possibility that more than one NTF may impact a portion of neurons that co-express more than one NTF receptor.

Embryonic and neonatal sympathetic and sensory neurons are dependent on NGF for survival. Therefore, NGF deprivation was initially thought to induce reactivation through apoptotic signaling. However, embryonic sympathetic neurons continue to mature in vitro, losing their dependence on NGF for survival after about three weeks in culture [28], and prevention of apoptosis with a pan-caspase inhibitor did not prevent HSV1 reactivation following NGF withdrawal [22]. Thus, reactivation does not occur simply through a “death” signal in the host neuron. Additional studies showed that NGF deprivation led to cessation of signaling through the tyrosine kinase TrkA and the PI3-K/Akt pathway, demonstrating the importance of continuous downstream signaling events to maintain HSV1 in a latent state [22]. Even transient disruption of the PI3-K/Akt pathway results in loss of downstream mTORC1 activity and subsequent HSV1 reactivation [35], demonstrating an active role in neuronal signaling events to maintain the latent state. GDNF and NTN activation of their preferred receptors, GFRα1 and GFRα2, result in a similar downstream signaling cascade, activating the RET tyrosine kinase and PI3-K/Akt pathway, as well as STATs, PLCγ/PKC/ERK, JNK/JUN, and β-catenin/TCF-4 pathways, all of which have been implicated in some aspect of HSV1 maintenance within cells. Activation of JNK, which can occur through various neuronal stressors including NGF deprivation and axotomy, was recently shown to be a requirement for triggering phase I of HSV1 reactivation, prior to changes in histone conformation or viral protein synthesis [36]. Additional factors have been reported to be requirements for full reactivation, including but not limited to cellular histone demethylases [37,38] and the viral transactivator VP16 [39]. Although further studies are necessary to identify the specific pathway through which NTN and GDNF maintain HSV1 and HSV2 in a latent state in sensory neurons, it is possible that the same signaling pathways identified for maintenance of HSV1 latency in embryonic neurons are also responsible for maintenance of latency in adult neurons. However, since latency is established in a diverse, heterogeneous population of adult neurons, different neurotrophic factors are required to maintain the signaling events within each neuronal subpopulation. Thus, signaling pathways may be similar or identical, but because the adult neuronal populations are responsive to different environmental signals, successful maintenance of HSV1 and HSV2 latency in their respective neuronal hosts requires different neurotrophic factors. Alternatively, different pathways may lead to reactivation in different types of neurons, which could account for the complex array of reported mechanisms and factors that control latency and reactivation. GDNF deprivation activates an alternative pathway in sympathetic neurons, compared to NGF deprivation [40], demonstrating that two different neurotrophic factors acting on the same neuron may also produce different responses within that cell. Latency is a dynamic state [41] and clearly a complex relationship between the host neuron and the virus. Given the recent characterization of the heterogeneity of discrete sensory neuronal populations by single-cell transcriptomics [42], we now have new tools available to more clearly define the specific types of neurons in which HSV1 and HSV2 establish latency and decipher events that lead to reactivation.

Loss of NGF or NTN signaling can occur during nerve injury, such as peripheral epithelial damage related to a sunburn or micro-abrasions, depriving the axon terminal of target-derived neurotrophic factors expressed in the epithelium. Loss of NGF or NTN signaling would induce HSV1 reactivation in NGF-responsive sympathetic neurons or NTN-responsive sensory neurons. GDNF, which maintains HSV2 latency in adult sensory neurons, is expressed in testes, ovaries, kidneys, and at lower levels in skin, identifying it as a target-derived neurotrophic factor, as well [43]. However, GDNF is also expressed by some peptidergic neurons within the sensory ganglia, transported anterograde to the central axon terminal, and released into the central nervous system where it can modulate nociceptor and interneuron signaling [44]. Therefore, disruption of GDNF signaling could potentially occur at the central axon terminal or at the peripheral terminal in the pelvic organs or epithelium, resulting in HSV2 reactivation in GDNF-responsive neurons. Different expression patterns of NTN in the skin and GDNF in pelvic organs, combined with establishment of HSV1 and HSV2 latency in specific populations of neurons responsive to these neurotrophic factors, could therefore be responsible for different reactivation and recurrent disease patterns for HSV1 and HSV2. While studies have shown that treatment with an anti-NGF antibody can induce reactivation in vivo and in explanted sensory ganglia, a combination of NTF effects is likely occurring. Treatment with anti-NGF antibody in rabbits resulted in viral shedding in tears [45]. Our results showing that NGF deprivation and treatment with anti-NGF antibody reactivates HSV1 from sympathetic neurons but not sensory neurons in vitro suggests that the administration of anti-NGF antibody in vivo may have induced HSV1 reactivation in the SCG, which innervates the lacrimal glands. Anti-NGF antibody treatment was also found to induce reactivation in explanted sensory ganglia [46]. Removal of the ganglia from latently infected mice results in loss of not only NGF support, but also other target derived NTFs, including GDNF and NTN. Our results show that HSV1 reactivation occurred from sensory neurons in response to NTN deprivation, not NGF deprivation, but NGF deprivation enhanced acute viral replication. Therefore, it is possible that NTN deprivation after axotomy of the latently-infected ganglia induced reactivation from NTN-responsive neurons and the presence of the anti-NGF antibody further enhanced replication following reactivation and viral spread to other neuronal subtypes in the intact explanted ganglion. The acceleration of viral mRNA accumulation in response to anti-NGF antibody in the media was detected 15–18 h post-explant [46], which would allow time for a full cycle of viral replication in neurons that rapidly respond to the reactivation stimulus (axotomy and loss of NTN support) and release of viral progeny, which could spread to other neurons or satellite glial cells.

**Figure 5:**
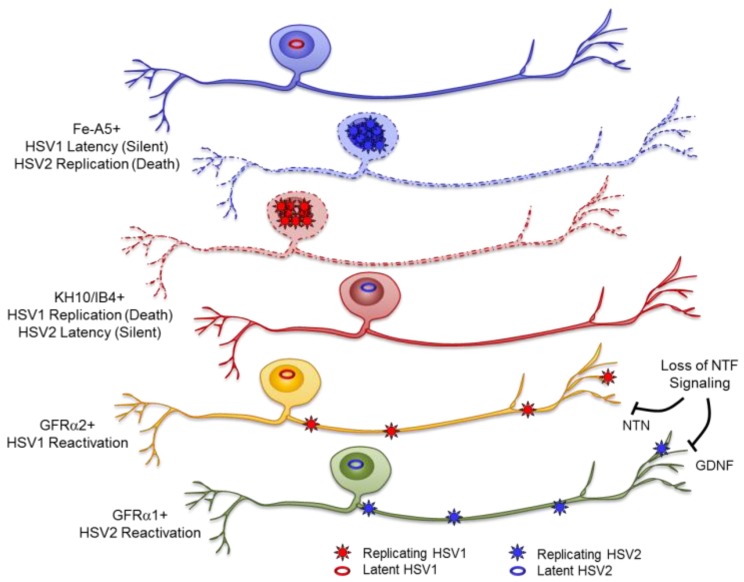
Model of neuron-specific outcome of infection with HSV1 and HSV2. HSV1 preferentially establishes latency in Fe-A5+ neurons but does not reactivate, replicates in KH10/IB4+ neurons and kills them, and reactivates from latency in GFRα2+ neurons in response to loss of NTN signaling. HSV2 replicates in Fe-A5+ neurons, preferentially establishes latency in KH10/IB4+ neurons but does not reactivate, and reactivates from latency in GFRα1+ neurons in response to loss of GDNF signaling.

Our results, combined with results from previous studies [22,23], show that loss of NGF signaling can induce reactivation of HSV1 from sympathetic neurons, regardless of their level of maturation. While GDNF does not fully support HSV1 latency in embryonic sympathetic neurons [22], loss of GDNF signaling in adult sympathetic neurons is just as effective at reactivating HSV1 as loss of NGF signaling. Even though adult sympathetic neurons are responsive to NGF and GDNF deprivation, resulting in reactivation of HSV1, the same stimuli fail to reactivate HSV2 in these neurons, demonstrating profound differences between HSV1 and HSV2 reactivation mechanisms. In contrast to embryonic sensory neurons, NGF deprivation has minimal impact on either HSV1 or HSV2 reactivation in adult sensory neurons. HSV1 and HSV2 reactivate from different populations of sensory neurons responsive to NTN and GDNF, respectively. Our results, combined with results from previous studies [5,6,7,9], support a model in which different populations of neurons support three different outcomes of HSV1 and HSV2 infection, and these neuronal populations differ for HSV1 and HSV2 (Figure 5). In IB4+ neurons, HSV1 replicates and kills the majority of these neurons that it enters; in Fe-A5+ neurons (a subset of NGF-responsive TrkA+ neurons), HSV1 preferentially establishes 50% of its latent sites but appears to be incapable of reactivating, so Fe-A5+ neurons represent a silent dead end for HSV1; in GFRα2+ neurons, HSV1 establishes latency but is competent for reactivation in response to loss of NTN signaling. Although many of the GFRα2+ neurons are within the IB4+ population, TrkA-/IB4- and some TrkA+ neurons also express GFRα2 (our results and [42]), suggesting a distinct neuronal population that supports HSV1 reactivation. In contrast, HSV2 replicates in Fe-A5+ neurons, preferentially establishes latency in IB4+ neurons, but is reactivation competent in GFRα1+ neurons in response to loss of GDNF signaling. Approximately 40% of adult sensory neurons are GFRα1+ (our results and [34]) many of which are within the IB4+ population, but GFRα1 is also expressed by some TrkA+ neurons, so it remains to be determined if reactivation occurs from IB4+/GFRα1+ or IB4-/GFRα1+ neurons. Therefore, appropriate neuronal populations must be considered to fully understand how HSV1 and HSV2 establish latency, and how the relationship changes between the host neuron and the latent HSV episome, resulting in viral reactivation and recurrent disease.

## 4. Materials and Methods

### 4.1. Virus Strains

HSV-1 strain 17+ was originally transferred from John Hay (SUNY Buffalo, Buffalo, NY, USA) and HSV-2 strain 333 from Gary Hayward (Johns Hopkins, Baltimore, MD, USA) to the Krause lab (FDA, Bethesda, MD, USA). Viruses were propagated in Vero cells (ATCC) and first passage stocks were transferred to the Margolis lab (UCSF, San Francisco, CA, USA). Viruses were propagated in Vero cells and first passage stocks were transferred to the Bertke lab (Virginia Tech, Blacksburg, VA, USA). Viruses were propagated in Vero cells and titrated in quadruplicate by plaque assay in Vero cells. Stock viruses were diluted in DMEM for inoculations.

### 4.2. Quantitation of HSV Viral Load and Gene Expression

Viral DNA was extracted from cultured neurons with Trisure reagent (Bioline), according to the manufacturer’s instructions. Quantitative PCR was performed on a Viia7 realtime PCR machine (Applied Biosystems), using iTaq universal probe mix (Biorad) and ZEN primer/probe sets (IDT) specific for HSV-1 or HSV-2 thymidine kinase (TK) gene: HSV-1 TK forward 5′-AAAACCACCACCACGCAACT-3′, reverse 5′-TCATCGGCTCGGGTACGTA-3′, probe 5′-TGGGTTCGCGCGACGATATCG-3′; HSV-2 TK forward 5′-taatgaccagcgcccagat-3′, reverse 5′-CGATATGAGGAGCCAAAACG-3′, probe 5′-ACAATGAGCACGCCTTATGCGGC-3′ [47,48]. All assays were normalized to 18s rRNA (Applied Biosystems) and reported as quantity of viral genome copies in 200 ng of total DNA.

### 4.3. In Vitro Infection

Trigeminal (TG) and superior cervical (SCG) ganglia were removed from 6 week old Swiss Webster mice and cultured on Matrigel-coated 8-well Lab-Tek II chamber slides (ThermoScientific), as previously described [5]. Briefly, ganglia were digested in papain, collagenase and dispase (Worthington), followed by mechanical trituration with a pipette. TG were passed through an OptiPrep (BD Biosciences) gradient to enrich for neurons; SCG were plated without the gradient step, since they contain minimal axonal debris in the cell suspension. Cells were washed and plated in Neurobasal A medium supplemented with 2% SM1 supplement (StemCell), 1% penicillin-streptomycin, L-glutamine, neurotrophic factors (NGF, GDNF, NTN, from PeproTech), and mitotic inhibitors (Life Technologies). Four days after plating, media was removed, neurons were inoculated with HSV-1 (strain 17+) or HSV-2 (strain 333), viruses were allowed to adsorb for one hour, and complete Neuro media (Neurobasal A, SM1, L-glutamine and neurotrophic factors, with no mitotic inhibitors) was added. Acute cultures were treated with specific neurotrophic factor-deprived media and their respective antibodies, and DNA was isolated 24 h post inoculation by qPCR. This time period was selected to allow for a complete viral growth cycle from those neurons that are rapidly responsive to the loss of NTFs, without providing a substantial delay for non-specific secondary or off-target effects to impact the viruses. TG (*n* = 11).

### 4.4. In Vitro Reactivation

Four days after plating, neurons were inoculated as described for in vitro infection. After the one hour adsorption period, cultures were treated with 300 μM acyclovir (ACV) in complete Neuro media to establish experimental latency in neuronal cultures. Seven days later, media was removed and replaced with fresh media, omitting ACV and specific neurotrophic factors and including the respective antibodies. DNA was isolated 24 h post-treatment for qPCR. TG (*n* = 19), SCG (*n* = 3).

### 4.5. Immunofluorescence

Neuronal cultures were fixed with 2% paraformaldehyde and immunostained for HSV antigens with polyclonal antisera (Dako). Cultures were also stained for neuronal markers TrkA (specific antibody, 1:250, Abcam), GFRα1 (specific antibody, 1:200, Abcam), GFRα2 (specific antibody, 1:250, Abcam), and IB4 (FITC or rhodamine conjugate, 1:500, Vector). Neurons were counted to determine the percentage of HSV-positive neurons for each neuronal marker or of total neurons. Infections were repeated three times in duplicate.

## Figures and Tables

**Table 1:** HSV1 and HSV2 behavior in different murine neuronal populations.

	HSV1	HSV2
**Replication**	KH10/IB4+	Fe-A5+
**Latency**		
Fe-A5+ neurons	43.3% ^1^	4.2% ^1^
KH10/IB4+ neurons	4.6% ^1^	47.2% ^1^
Other neurons	52.1%	48.6%
**Reactivation**		
Fe-A5+ neurons	??	No ^2^
KH10/IB4+ neurons	No ^2^	??
Other neurons	Unknown type	Unknown type

^1^ Average of percentages compiled from all wildtype viruses published [6,7,10]. ^2^ Most die during lytic infection so few neurons harbor latent HSV from which the viruses can reactivate.

## Supplementary Files

Supplementary File 1
