# Supplementary figures and images for: Neurotrophic Factors NGF, GDNF and NTN Selectively Modulate HSV1 and HSV2 Lytic Infection and Reactivation in Primary Adult Sensory and Autonomic Neurons
Source: Pathogens. 2017 Feb 7;6(1):5. doi: 10.3390/pathogens6010005 (PMC5371893; doi:10.3390/pathogens6010005)

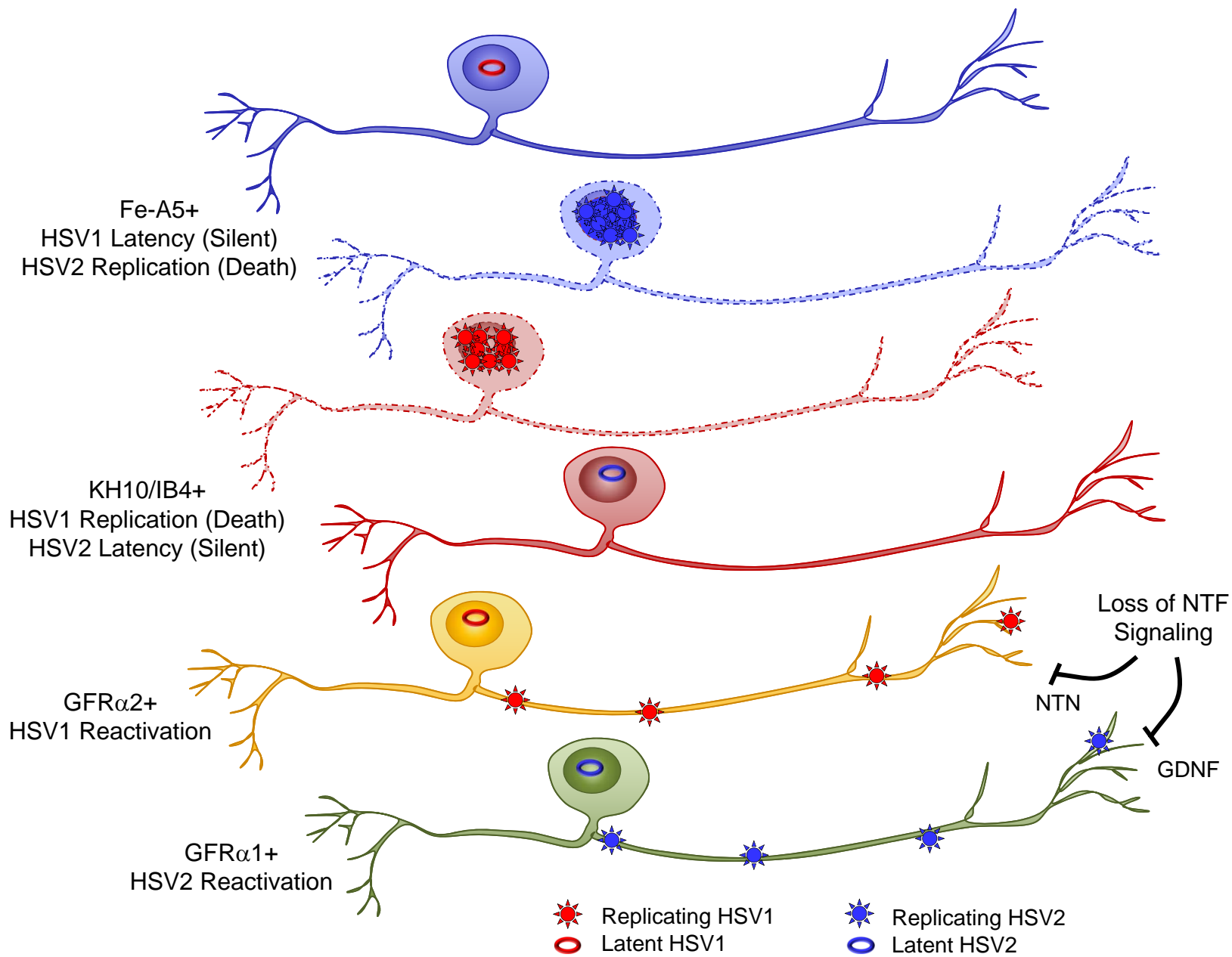

Supplement: Supplementary File 1 [file pathogens-06-00005-s001.pdf]
